# Topical Mitomycin-C versus Subconjunctival 5-Fluorouracil for Management of Bleb Failure

**Published:** 2011-04

**Authors:** Mohammad Pakravan, Arezoo Miraftabi, Shahin yazdani, Nasim Koohestani, Mehdi yaseri

**Affiliations:** 1 Ophthalmic Research Center, Shahid Beheshti University of Medical Sciences, Tehran, Iran; 2 Eye Research Center, Rassoul Akram Hospital, Tehran University of Medical Sciences, Tehran, Iran; 3 Department of Epidemiology and Biostatistics, School of Public Health, Tehran University of Medical Sciences, Tehran, Iran

**Keywords:** Glaucoma, Mitomycin-C, 5-Fluorouracil, Trabeculectomy

## Abstract

**Purpose:**

To compare the efficacy and safety of topical mitomycin-C (MMC) drops with that of subconjunctival 5-fluorouracil (5-FU) injections for management of early bleb failure after trabeculectomy or combined phacoemulsification and trabeculectomy with posterior chamber intraocular lens implantation (PT+PCIOL).

**Methods:**

In a randomized comparative study, 37 eyes of 37 patients with impending early bleb failure received MMC 0.02% eye drops for 2 or 4 weeks (19 eyes) or subconjunctival 5-FU injections, 5 mg per dose (18 eyes). Complete success was defined as 5 < IOP ≤ 18 mmHg without medications.

**Results:**

Baseline characteristics were comparable between the study groups. However, there were more cases of combined PT+PCIOL in the MMC group [11 (57.9%) eyes versus 3 (16.7%) eyes, P = 0.017]. Mean preoperative IOP was 20.5±8.85 mmHg in the MMC group and 25.82±11.35 mmHg in the 5-FU group (P = 0.129), which was decreased to 13.2±6.1 and 10.6±4.8 mmHg respectively after 12 months (P = 0.159). There was no significant difference between the study groups in terms of bleb extent (P = 0.170), height (P = 0.178) or vascularity (P = 0.366). At the end of the study, complete success was achieved in 13 eyes (68.4%) in the MMC group and 14 eyes (77.8%) in the 5-FU group (P = 0.714). The survival of success at 8 months (median follow-up) was 89.5% and 86.5% in the MMC and 5-FU groups respectively; the number of glaucoma medications (P = 0.707) and best-corrected visual acuity (P = 0.550) were also comparable. Complication rates were similar in the study groups (P = 0.140).

**Conclusion:**

Topical MMC 0.02% has comparable safety and efficacy to subconjunctival 5-FU injections for management of early bleb failure. Topical MMC 0.02% drops are more convenient and can be initiated first, while 5-FU injections may be reserved for eyes with an insufficient response to topical MMC.

## INTRODUCTION

Despite the adoption of several surgical procedures since the introduction of trabeculectomy in 1961, it remains the most common glaucoma operation.^1^ In this procedure, after dissecting a partial thickness scleral flap, a fistula is created between the anterior chamber and the subconjunctival space, and a bleb is formed under the conjunctiva and Tenon’s capsule providing aqueous filtration outside the globe.^2^ Gentle intraoperative tissue handling and the use of steroids and anti-fibrotic agents have been suggested to maintain the patency of this conduit; however, this goal is not always attained and postoperative inflammation and scar formation may lead to early bleb failure during the first few months after surgery.^2^,^3^ Increased vascularity and reduction in bleb height and area, with or without an increase in intraocular pressure (IOP), constitute the early signs of bleb failure. Prompt measures are to be taken before permanent adhesions develop between the conjunctiva and episclera. The use of frequent topical or systemic corticosteroids, ocular massage, and the removal of releasable sutures or laser suture lysis are initial measures for management of this situation. Persistent bleb vascularization may be a poor prognostic sign for bleb survival and requires prompt intervention.^1^,^4^

Adjunctive antimetabolites are used postoperatively for further reduction of subconjunctival fibrosis, which is especially important in eyes at high risk of failure. Postoperative subconjunctival injections of 5-fluorouracil (5-FU) can retard bleb fibrosis and enhance filtration, but are inconvenient for both the patient and surgeon, furthermore they entail complications such as corneal and conjunctival epithelial toxicity, corneal ulcers, and inadvertent intraocular penetration of 5-FU. These injections are painful and impractical for young children and patients with limited cooperation.^3^ Local sponge applications of mitomycin-C (MMC) and subconjunctival MMC injections with needling have also been used to address early bleb failure, but these types of application are also cumbersome and lead to complications.^5^–^7^

The purpose of this study was to compare the safety and efficacy of postoperative application of topical MMC drops with that of subconjunctival 5-FU injections for management of early bleb failure.

## METHODS

This randomized comparative study investigated the safety and efficacy of topical MMC vs. subconjunctival 5-FU for management of impending early bleb failure following trabeculectomy or combined phacoemulsification and trabeculectomy with posterior chamber intraocular lens implantation (PT+PCIOL) in a consecutive series of patients from June 2007 to July 2009. The study was approved by the ethics committee of the Ophthalmic Research Center and was registered at www.ClinicalTrials.gov (NCT 00644215), according to the standards set by the International Committee of Medical Journal Editors and the World Health Organization. Written informed consent was obtained from all patients prior to enrollment after providing an explanation about the study goals and procedures.

All procedures were performed by three surgeons (MP, SY, and AM). A fornix-based conjunctival flap was created, and after scleral flap dissection, at least five sponges soaked with MMC 0.02% were placed under the conjunctiva for two minutes for trabeculectomy alone and three minutes for phacotrabeculectomy, followed by copious irrigation with balanced salt solution. After anterior chamber paracentesis, the block was removed and a peripheral iridectomy was performed. The scleral flap was secured with two releasable 10-0 nylon sutures and the conjunctiva was repaired with 10-0 nylon sutures.

Eyes with injected blebs and decreased bleb height, with or without increased IOP, no later than one month after trabeculectomy or combined PT+PCIOL which did not respond to frequent topical corticosteroids and massage or releasable suture removal, and at the same time had a patent internal block on gonioscopy, were randomly assigned to the study groups according to a computer generated randomization scheme. MMC 0.02% drops (Kyowa Hakko Kogyo Co., Tokyo, Japan) were prepared using artificial tears (Sno tears; Bausch & Lomb, London, England) as the vehicle and were applied four times a day for two weeks and repeated for another two-week period one week after completion of the initial course if signs of failure persisted. To reduce systemic absorption of MMC, patients were instructed to occlude their puncti by digital compression for 5 minutes. Because of ethical obligations, 5-FU injections were initiated according to surgeon’s discretion if signs of failure showed an insufficient response to MMC drops at any follow up examination.

The subconjunctival 5-FU group received 5 milligrams of 5-FU (Ebewe Pharma, Unterach, Austria) once daily close to the bleb, until a desirable reduction in signs of bleb failure appeared or up to a total dose of 65 mg. In eyes developing complications, such as filamentary keratitis, further injections or MMC drops were avoided, however in cases with superficial punctate keratitis, the drugs were continued with caution.

Recorded data included age, sex, type of glaucoma, number and duration of antiglaucoma medications, and previous ocular procedures. Main outcome measures included IOP and bleb morphology according to the Indiana Bleb Appearance Grading Scale (IBAGS).^8^ Other outcome measures included success rate, number of glaucoma medications, best-corrected visual acuity (BCVA), and complications. Complete success was defined as 5 < IOP ≤ 18 mmHg without medications and qualified success was defined as the same range of IOP with one or two topical glaucoma medications. For the purpose of bleb grading, slitlamp photographs were taken. IBAGS was used for morphological classification of filtering blebs.^8^ Based on this grading system, bleb height is assessed by the vertical width of the conjunctival flap over the scleral surface and classified as H_0_: flat bleb, H_1_: low elevation, H_2_: moderate elevation, and H_3_: high elevation. Bleb extent represents the dimensions of the filtering bleb and is divided into four grades including E_0_: no visible or less than 1 clock hour bleb extension, E_1_: extension equal to or greater than 1 clock hour but less than 2 clock hours, E_2_: extension equal to or greater than 2 clock hours but less than 4 clock hours, and E_3_: extension equal to or greater than 4 clock hours. Bleb vascularity is assessed by examination of superficial and deep vessels and is divided into 5 grades including V_0_: white avascular bleb, V_1_: cystic avascular bleb, V_2_: mild vascularity, V_3_: moderate vascularity, and V_4_: extensive vascularity. Since only injected blebs without leakage (S_0_) were enrolled in the study, we did not employ Seidel test assessment.

Examinations and slitlamp photography were performed 1, 7 and 14 days, and 1 and 3 months after enrollment. Complete eye examinations were performed every three months thereafter. Data of eyes with at least three months of follow up were used for statistical analysis; however, failed cases during this period were included. Two masked glaucoma specialists evaluated the slitlamp photographs and classified bleb configuration based on IBAGS scores. An independent biostatistician was appointed to evaluate the outcome measures. Data were modeled using the SPSS software package version 15 (SPSS Inc., Chicago, IL, USA). Data were compared by t-test, Mann-Whitney, Chi-square, and Fisher Exact tests. To adjust for baseline effect, analysis of covariance (ANCOVA) was utilized. P-values less than 0.05 were considered significant.

## RESULTS

A total of 37 eyes of 37 patients, including 29 male (78.5%) and 8 female (21.5%) subjects, with mean age of 55.2 ± 23.4 (range, 9 to 85) years were included in the study; 19 eyes were randomized to receive topical MMC drops while 18 eyes underwent 5-FU injections. Patients were followed for a mean period of 11.5 ± 8.0 (median, 8) and 10.9 ± 5.4 (median, 11) months in the MMC and 5-FU groups, respectively (P = 0.589). Table 1 summarizes basic and demographic characteristics of the patients. No significant difference was observed between the study groups in terms of age, sex, type of glaucoma, BCVA, mean preoperative IOP, cup to disc ratio, duration of glaucoma medication use, and previous ocular or glaucoma procedures. One difference was in the mean number of preoperative glaucoma medications which was 2.5 ± 0.7 in the MMC group vs. 3.2 ± 0.8 in the 5-FU group (P = 0.012). Surgical complications were not significantly different between the groups (P = 0.605). By chance, there were more combined PT+PCIOL procedures in the MMC group as compared to the 5-FU group [11 (57.9%) vs. 3 (16.7%) eyes, P = 0.017]. The mean interval between surgery and intervention was 7.9 ± 5.1 and 9.7 ± 5.6 days in the MMC and 5-FU groups respectively (P = 0.345). Thirteen (68.4%) eyes in the MMC group received two cycles of therapy, the remaining patients received only one cycle of MMC drops for two weeks. The mean total dose of 5-FU was 19.4 ± 17.0 mg (range, 5 to 65 mg) administered with a mean number of 2.8 (range, 1 to 13) injections.

**Figure 1:**
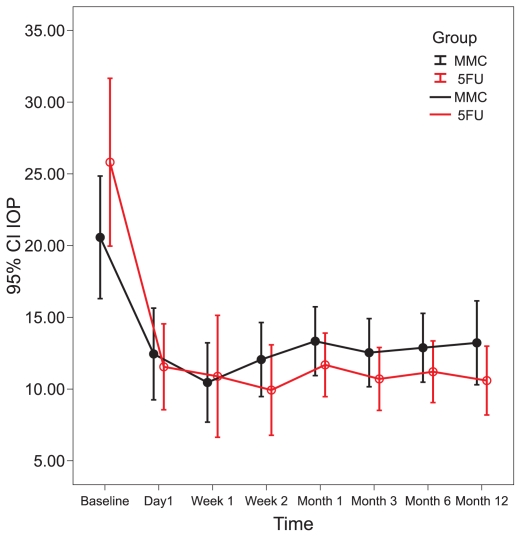
Change in intraocular pressure from baseline in the MMC and 5-FU groups.

**Figure 2:**
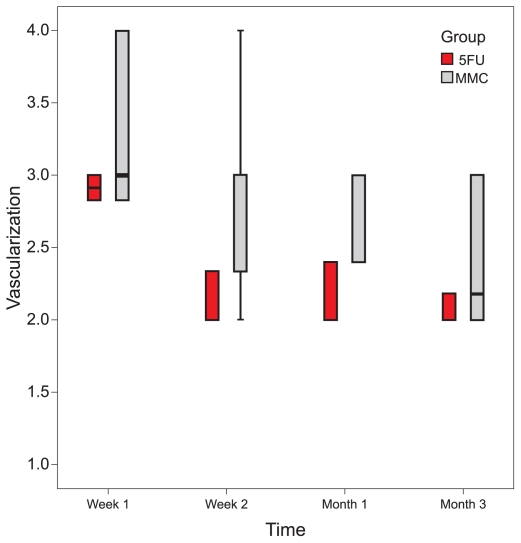
Changes in bleb vascularity from baseline in the MMC and 5-FU groups.

**Figure 3:**
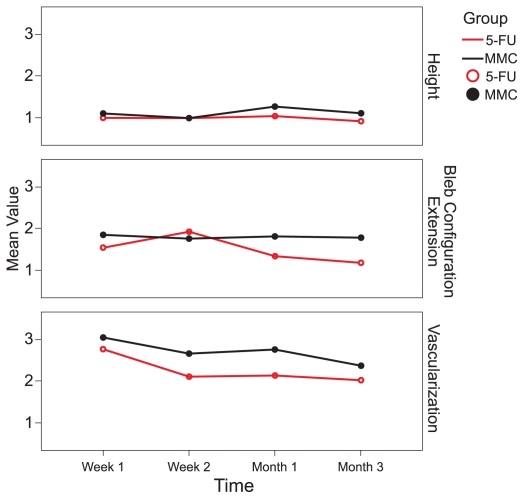
Changes in bleb appearance from baseline in the MMC and 5-FU groups.

Intraocular pressure was significantly reduced from baseline in both study groups at 1 and 2 weeks and 1, 3, 6, and 12 months (all P-values ≤ 0.01). No significant difference was observed between the study groups in terms of IOP at any interval (all P-values > 0.25) (Table 2 and Fig. 1). There were no significant differences in bleb height and extent between the two groups (bleb extent: P = 0.170, height: P = 0.178, vascularity: P = 0.366). In the MMC group there was a borderline decrease in bleb vascularity after one week (P = 0.05), and a significant decrease after two weeks, and one and three months (P < 0.05 for all comparisons). Bleb vascularity was decreased significantly in the 5-FU group after one and two weeks, and at one and three months (P < 0.05 for all comparisons; Fig. 2). The study groups were comparable in terms of bleb morphology up to 3 months after intervention (P = 0.380; Fig. 3).

**Figure 4:**
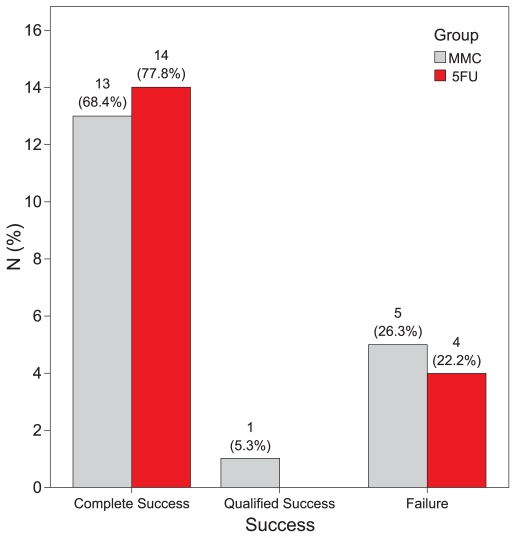
Success and failure in the MMC and 5-FU groups.

**Figure 5:**
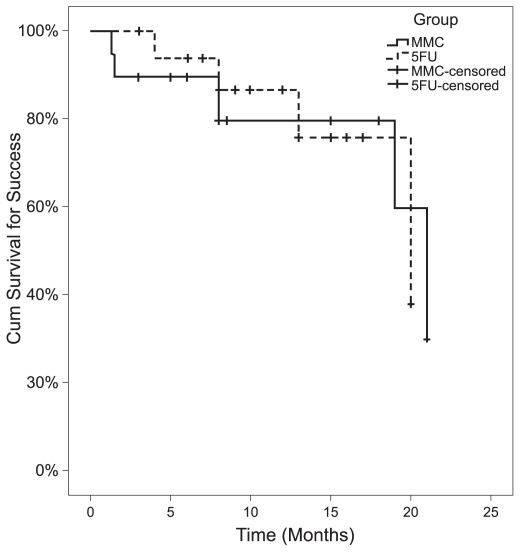
Kaplan-Meier survival curves for cumulative success rates in the MMC and 5-FU groups.

In the MMC group, complete and qualified success was achieved in 13 (68.4%) and 1 (5.3%) eyes, respectively; three eyes demonstrated failure, two of which had an IOP higher than 18 mmHg and one eye developed IOP of 5 mmHg without hypotony maculopathy. In the 5-FU group, 14 (77.8%) eyes achieved complete success and four eyes showed failure due to high IOP. There was no significant difference in success rates between the study groups (P > 0.999; Fisher Exact test, for both success rates simultaneously; Fig. 4). Success rates were comparable in the study groups throughout follow-up as demonstrated by Kaplan-Meier analysis (P = 0.974; Fig. 5). The median time for survival was 21 months (95% CI: 18–24) in the MMC group and 20 months (95% CI: 10–30) in the 5-FU group. Six eyes in the MMC group received additional 5-FU injections according to IOP or bleb appearance at a mean dose of 10.7 ± 10.1 mg (range, 5 to 65 mg), 35.5 ± 11.8 days after surgery. Four of these eyes had undergone combined PT+PCIOL and two (10.5%) demonstrated failure despite 5-FU injections.

**Figure 6:**
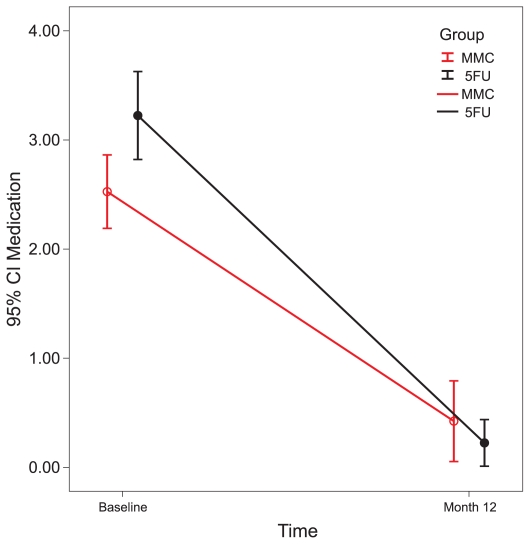
Number of medications in each group.

In both groups, the number of glaucoma medications decreased significantly. In the MMC group it was reduced from 2.5 ± 0.7 preoperatively, to 0.4 ± 0.7 after 12 months. Corresponding figures for the 5-FU group were 3.2 ± 0.8 and 0.2 ± 0.4 (P < 0.001 for both groups; Fig. 6). Final BCVA was comparable between the study groups and was 0.83 ± 0.51 logarithm of minimum angle of resolution (LogMAR) in the MMC and 0.73 ± 0.55 LogMAR in the 5-FU group (P = 0.550, ANCOVA). The most common complication related to MMC was punctate epithelial keratopathy, observed in 6 eyes (31.5%), and the most common complication of 5-FU injections was filamentary keratitis, in 7 eyes (38.9%). Complication rates were comparable between the study groups (P = 0.140; Table 3).

## DISCUSSION

In the present study we compared the safety and efficacy of MMC drops with 5-FU injections for management of early bleb failure after filtering surgery. We found MMC drops to be comparable to 5-FU injections in terms of IOP, bleb appearance, success rate, number of glaucoma medications, visual outcome, and overall complications. Complete success was similar in both groups and was achieved in 68.4% and 77.8% of eyes in the MMC and 5-FU groups, respectively.

Adjunctive antimetabolites are used postoperatively to reduce subconjunctival fibrosis, which is especially important in eyes at high risk of failure after trabeculectomy. Antimetabolites such as 5-FU and MMC inhibit fibroblast proliferation and scar tissue formation.

5-FU is a pyrimidine analog that acts by competitive inhibition of the enzyme thymidylate synthetase and is cell-cycle specific, making it more toxic to replicating cells.^9^ It can be used as a soak during surgery or as subconjunctival injections after the procedure. Several studies have demonstrated the benefits of 5-FU in glaucoma surgery.^10^–^13^

MMC is an antibiotic isolated from Streptomyces caespitosus and is a hundred times more potent than 5-FU and also toxic to vascular endothelium. Several studies have revealed the efficacy of intraoperative MMC in increasing the success rate of trabeculectomy.^14^,^15^ It has been demonstrated that MMC penetrates subconjunctival tissues after application over an intact conjunctiva.^16^ Topical MMC drops have been used effectively for the treatment of corneal intraepithelial neoplasia.^17^–^19^ In addition, there are limited studies reporting that the application of MMC with a sponge can improve IOP control after trabeculectomy in rabbits and humans.^7^,^8^,^20^

In a study by Mietz et al^7^, postoperative surface application of MMC was compared with intraoperative application. Hypotony was more frequent in the group with intraoperative application, in which the only case of hypotony maculopathy occurred. There was also a tendency toward lower IOP in this group. The rates of loss of visual acuity exceeding two lines and failure were also higher in this group.

Khong and Muecke^21^ applied topical MMC 0.04% in 100 eyes of 91 patients with ocular surface neoplasia and reported the most common complication to be allergic reactions with marked pruritus, periocular erythema, and redness in 34% of cases. Punctal stenosis and epiphora developed in 14% of their patients. None of these complications were observed in any of our patients, which may be due to the lower concentration of MMC used in the current study (0.02%). Furthermore, we limited MMC drops to two cycles of two weeks’ duration with one week of discontinuation in between. Of our patients, 68.4% were treated with two cycles of therapy, the remaining patients received only one cycle. Khong and Muecke observed no allergic reactions during the first course of MMC application in any of their cases; it is possible that delayed hypersensitivity could be responsible for the reported allergic reactions.^21^

Araie et al^13^ used subconjunctival 5-FU in 263 patients during the first postoperative days after trabeculectomy. The mean 5-FU dose was 36 ± 19.5 mg in eyes with primary open angle glaucoma, 49.5 ± 18 mg for refractory glaucoma, and 36.5 ± 20 mg for secondary glaucoma. We used a mean dose of 19.4 ± 17.0 mg in our study. This lower dose is probably due to the intraoperative application of MMC in all eyes as a routine practice in our study. Araie et al reported corneal epithelial defects in 36.8% of eyes in their series following 5-FU injections. We observed filamentary keratitis in 38.9% of eyes in the 5-FU group and only 5.26% of those receiving MMC. Superficial punctate keratopathy was observed in 26.3% of eyes receiving MMC drops which responded rapidly to lubricants.

In our study, the mean interval between initiation of the antimetabolites and surgery was 8.6 ± 5.2 days in the MMC group vs. 9.9 ± 0.7 days in the 5-FU group. This is different from studies by Van Buskirk^12^ and Araie et al^13^, in which 5-FU was initiated on the first day after surgery. Mietz and Krieglstein^5^ applied topical MMC 0.05 mg/cc for 5 minutes over the filtering bleb with a sponge for 3 days postoperatively. This approach reduced IOP without increasing complications. Reinthal et al^11^ started 5-FU injections 4.6 ± 8.5 days postoperatively in 172 eyes of 171 patients who had undergone trabeculectomy. The total 5-FU dose ranged from 5 to 56 mg which was similar to our range (5 to 65 mg).

By chance, there were more combined PT+PCIOL procedures in the MMC group and for this reason eyes in the MMC group were at higher risk for bleb failure. Following inadequate response to therapy which could have been due to a larger number of high-risk cases in this group, and according to surgeon’s discretion, 5-FU injections were initiated in six eyes in the MMC group. Four eyes in this group achieved an IOP between 6 and 18 mmHg without medication, while two eyes demonstrated failure despite 5-FU injections. We observed complete success rates of 68.4% and 77.8% in the MMC and 5-FU groups, respectively, which are similar to results of the Mietz and Krieglstein^5^ study, in which 84.6% of the patients who received postoperative MMC applications with a sponge, achieved complete success after 6 months (IOP < 22 mmHg without medication).

One of the limitations of our study is the duration of follow up, even though 12 months seems adequate to compare the response of early bleb failure to either treatment protocol. The disproportionate distribution of eyes with combined surgery into the study groups is another limitation of this study.

In this study, we observed comparable safety and efficacy for MMC drops in comparison with 5-FU injections for management of early post-trabeculectomy bleb failure. IOP, final bleb morphology, success rates, final number of glaucoma medications, and visual outcomes were comparable. Moreover, the rate of complications was also similar (P = 0.140). Postoperative application of MMC drops entails advantages such as convenience for both the patient and the physician, as compared to subconjunctival 5-FU injections. In eyes showing signs of early bleb failure following filtering surgery and when conservative measures, such as frequent topical or systemic corticosteroids, ocular massage, and removal of releasable sutures or laser suture lysis are not adequate, topical MMC 0.02% drops can be initiated first, reserving the cumbersome 5-FU injections for eyes in which signs of failure show an insufficient response to MMC drops.

## Figures and Tables

**Table 1:** Basic and demographic characteristics of patients in each study group

Variable	MMC (n=19)	5-Fu (n=18)	P-value
Age (years)	61.7 ± 18.9	48.4 ± 25.5	0.082†

Male sex [N(%)]	17 (89.5)	12 (66.7)	0.124‡

Baseline BCVA (LogMAR)	1.18 ± 0.64	0.82 ± 0.64	0.097†

Glaucoma subtype [N(%)]			0.192‡
POAG	5 (26.3)	2 (11.1)	
	
PACG	7 (36.7)	5 (27.8)	
	
Congenital	0 (0)	5 (27.8)	
	
PXF	2 (10.5)	3 (16.7)	
	
Uveitic	2 (10.5)	1 (5.6)	

Pseudophakic	3 (15.8)	2 (11.1)	

Previous surgeries (N)	1.0 ± 1.2	0.7 ± 1.0	0.443§

Duration of medications before surgery (years)	5.6 ± 7.2	6.0 ± 9.8	0.982§

Preoperative medications (N)	2.5 ± 0.7	3.2 ± 0.8	0.012§

Lens status [N(%)]			0.022*
Phakic	6 (31.6)	13 (72.2)	

Pseudophakic	13 (68.4)	5 (27.8)	

Preoperative IOP (mmHg)	20.6 ± 8.8	25.8 ± 11.4	0.129†

Cup/disc ratio	87.2 ± 13.6	83.1 ± 17.1	0.424†

Interval between surgery and trial (days)	7.9 ± 5.1	9.7 ± 5.6	0.265§

Type of operation [N(%)]			0.017‡
PT+PCIOL	11 (57.9)	3 (16.7)	
	
Trabeculectomy	8 (42.1)	15 (83.3)	

† t-test;

‡ Fisher Exact test;

§ Mann-Whitney test;

* chi-square test

MMC, mitomycin-C; 5-FU, 5-fluorouracil; N, number; BCVA, best-corrected visual acuity; POAG, primary open angle glaucoma; PACG, primary angle closure glaucoma; PXF, pseudoexfoliation; IOP, intraocular pressure; PT+PCIOL, combined phacoemulsification and trabeculectomy with posterior chamber intraocular lens implantation

**Table 2:** Intraocular pressures and changes from baseline in the two groups

		Group			
		MMC (n=19)	5-Fu (n=18)	95% CI of Difference	P-value
Baseline IOP	(mean ± SD)	20.6 ± 8.8	25.8 ± 11.4	−12.1 – 1.61	0.129†

First Day	(mean ± SD)	12.4 ± 6.4	11.5 ± 6.0	−3.3 – 5.1	0.671†
	Change from baseline	−7.3 ± 10.8	−14.4 ± 13.6	−1.4 – 15.4	0.748*
	(change %)	−27.0	−46.3		
	P within group†	0.010	<0.001		

First Week	(mean ± SD)	10.5 ± 5.3	10.8 ± 8.0	−5.1 – 4.3	0.865†
	Change from baseline	−9.7 ± 11.0	−16.4 ± 11.9	−1.5 – 15.1	0.939*
	(change %)	−36.3	−56.7		
	P within group†	0.002	<0.001		

Second Week	(mean ± SD)	12.1 ± 5.0	9.9 ± 6.3	−1.8 – 6.05	0.282†
	Change from baseline	−8.1 ± 8.6	−15.9 ± 12.1	0.42 – 15.1	0.181*
	(change %)	−32.4	−56.4		
	P within group†	0.001	<0.001		

First Month	(mean ± SD)	13.3 ± 4.8	11.7 ± 4.3	−1.5 – 4.8	0.301†
	Change from baseline	−7.6 ± 9.2	−14.1 ± 13.4	−1.5 – 14.3	0.369*
	(change %)	−26.5	−43.9		
	P within group†	0.003	0.001		

Third Month	(mean ± SD)	12.5 ± 5.0	10.7 ± 4.4	−1.3 – 4.9	0.251†
	Change from baseline	−8.1 ± 8.9	−15.1 ± 12.3	−0.1 – 14.3	0.196*
	(change %)	−30.7	−52.0		
	P within group†	0.001	<0.001		

Sixth Month	(mean ± SD)	12.9 ± 5.0	11.2 ± 4.3	−1.5 – 4.8	0.285†
	Change from baseline	−7.7 ± 8.9	−14.8 ± 12.1	−0.1 – 14.2	0.189*
	(change %)	−29.0	−50.4		
	P within group†	0.001	<0.001		

Twelfth Month	(mean ± SD)	13.2 ± 6.1	10.6 ± 4.8	−1.1 – 6.3	0.159†
	Change from baseline	−7.4 ± 10.4	−15.4 ± 12.1	−0.4 – 15.6	0.143*
	(change %)	−25.4	−52.7		
	P within group†	0.006	<0.001		

† Unadjusted P-value based on t-test;

* Adjusted P-value for baseline based on analysis of covariance

MMC, mitomycin-C; 5-FU, 5-fluorouracil; CI, confidence interval; IOP, intraocular pressure; SD, standard deviation

**Table 3:** Complications of interventions

	Group		
Complications	MMC (n=19)	5-Fu (n=18)	P-value
Filamentary keratitis	1 (5.3%)	7 (38.9%)	0.016*
Punctate epithelial keratitis	6 (31.5%)	2 (11.1%)	0.232*
Subconjunctival hemorrhage	0 (0.0%)	2 (11.1%)	0.230*
No complication	12 (63.2%)	7 (38.9%)	0.140†

* Fisher Exact test;

† chi-square

MMC, mitomycin-C; 5-FU, 5-fluorouracil
